# Treatment of high risk human papillomavirus infection in low grade cervical squamous intraepithelial lesion with mild local thermotherapy: Three case reports

**DOI:** 10.1097/MD.0000000000021005

**Published:** 2020-07-02

**Authors:** Yang Yang, Lan Zhang, Ruiqun Qi, Wei Huo, Xiaodong Li, Xin Wu, Hongduo Chen, Xing-Hua Gao

**Affiliations:** a Department of Dermatology, The First Hospital of China Medical University and Key Laboratory of Immunodermatology, Ministry of Health and Ministry of Education, National Joint Engineering Research Center for Theranostics of Immunological Skin Diseases, Liaoning; b Department of Dermatology, Central Hospital Affiliated to Shenyang Medical College; c Department of Obstetrics and Gynecology, The First Hospital of China Medical University, Shenyang, China.

**Keywords:** cervical intraepithelial neoplasia, human papillomavirus, hyperthermia, thermotherapy

## Abstract

**Introduction:**

Mild local hyperthermia at 44°C has been proven efficacious in the treatment of cutaneous warts induced by human papillomavirus (HPV), while its effect on cervical intraepithelial neoplasia (CIN) caused by high risk type of HPVs has not been reported.

**Patient concerns:**

Three patients with low grade CIN and positive high risk HPV types (HPV 16, 31, 52, 56, 58) are reported in this study.

**Diagnosis:**

The diagnosis was based on identification of HPV types and abnormal cytological findings.

**Interventions:**

The 3 patients were treated with local hyperthermia from ceramic heating (surface temperature, 44°C) to cervix. The treatment was delivered once a day for 3 consecutive days, plus two similar treatments 10 ± 3 days later, with each session lasting 30 minutes. HPV and cytology test were performed 3 months thereafter.

**Outcomes:**

All the 3 patients recovered to normal cytological findings. Two of the patients were negative for HPV, the remaining patient with pre-treatment HPV 56 and 58 positivity changed to HPV58 positive alone.

**Conclusion:**

This pilot observation inspires that mild local hyperthermia be recommended as a new method in the treatment of CIN patients with persistent HPV infection, once validated by qualified RCT.

## 1 Introduction

Cervical cancer is the fourth most commonly diagnosed female cancer worldwide, accounting for 7.5% of all female cancer deaths, especially in the less developed or developing regions, where more than 87% cancer deaths were recorded.^[1]^ Persistent high risk human papillomavirus (HPV) infection has been closely related to the onset and development of cervical dysplasia and cervical cancer. Currently, preventative vaccines, a bivalent, a quadrivalent and a 9-valent vaccine are available to protect against HPV infection, however there still remains quite a number of high-risk HPV types without vaccines. Previously infected women by high-risk HPVs could not benefit from the available vaccine and their chance to develop malignant transformation is unlikely to change. High-risk HPV infection remains a major therapeutic challenge nowadays.

Artificial elevated local temperature (exogeneous hyperthermia) has been used to treat infectious diseases and neoplasms, with varied efficacy.^[2]^ Our previous study demonstrated that hyperthermia at 44°C attained about 50% cure rate in the treatment of benign skin neoplasia caused by low risk HPVs, while this non-destructive method has not yet been applied in conditions by high risk HPVs.^[3]^ Herein, we presented 3 cases with cervical low grade squamous intraepithelial lesion (LSIL) with presence of high-risk HPVs, who responded favourably to local hyperthermia.

## 2 Case presentation

Three patients underwent a routine uterine cervical cancer screening with no other discomfort, and cytosmear indicated the existence of high-risk HPV and atypical squamous epithelial cells, but colposcopic examination showed no obvious abnormity. They were referred for treatment in our institution. Informed consent was obtained for publication of the case. This study was approved by the Ethical Committee of China Medical University ([2017]2016-207-2).

### 2.1 Method

**Figure 1:**
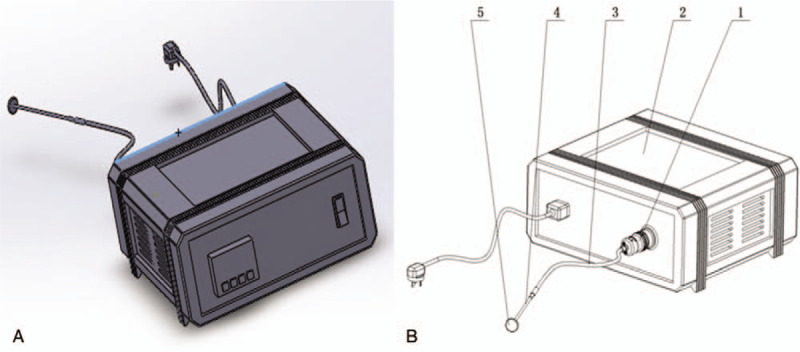
Structure of the main unit of the patented hyperthermia device. (A) The front pattern diagrams of the device. (B) The back structure and part names. 1. Removable plug: the plug makes it easy to remove the device; 2. Temperature control unit: this is the device component used to control temperature; 3. Rubber hose: metal bar with heating case is connected here. 4. Metal bar: this is the part inserted into vagina, serving as a thermosensor. 5. Applicator: the applicator can be used to clinging to the cervical opening in which alternating temperature is generated.

We used a patented local hyperthermia device (patent no. ZL 201620080552.2, China Medical University), which was approved by the Ethics Committee of China Medical University. The heat was generated from an energy source of ceramic heating element, which was delivered through a metal disk (2–3 cm in diameter) to the whole uterine cervix with direct contact, irrespective of the lesion location (Fig. 1). Output heating temperature was set at 44°C, and each session of treatment lasted 30 minutes. Patients were treated once daily for 3 consecutive days, and a week later, 2 more consecutive treatments were performed. Follow-up visit was scheduled 3 months after the treatment.

#### 2.1.1 Case 1

A 25-year-old married Chinese woman was admitted to our department. She was diagnosed with LSIL based on cytology findings 2 month ago. Her cervical cytosmear was positive for HPV16 and 58, tested by flow-through hybridization method. Cyto-immunohistochemical staining revealed p16 and Ki-67 positive cells, the percentage of the latter was greater than 25%. She was otherwise healthy except a 4-year history of eczema and 1-year history of leukoplakia vulvae. Three months after completion of treatment, her HPV (Scraping) test was negative and the cytology examination was normal.

#### 2.1.2 Case 2

A 49-year-old Chinese woman was diagnosed with LSIL 1 year and a half ago. Her cervical cervical cytosmear was positive for HPV31 and 56, tested by flow-through hybridization method. The patient received a combination of topical application of recombinant interferon (IFN) а-2b and subcutaneous injection of 1.6 mg thymalfasin, twice a week for 1 year, when she recovered with normal cytology and negative HPV test. Unfortunately, she was reinfected by HPV51 and 58, and was diagnosed with LSIL by cervical cytosmear examination 1 month ago. She also had a 4-year history of hepatic adipose infiltration. 3-month after the treatment, HPV (Scraping) test and the cytology examination displayed normal.

#### 2.1.3 Case 3

A 33-year-old married woman was diagnosed cervical intraepithelial neoplasia at grade 1 by pathological examination for a year. At the time, her cervical cytosmear was positive for HPV52, 58 and 16, and cyto-immunohistochemical staining presented positive p16 and the percentage of Ki-67 positive cells was greater than 20%. The patient was treated with topical recombinant IFNа-2b accompanied with injection of 20 mg thymopentin every other day for half a year. She was identified as HPV52 and 58 positive with a cervical cytological result of LSIL 1 month ago.

Three months after completion of hyperthermia treatment, she remained HPV58 positive and the cytology examination revealed normal (Table 1).

**Table 1:**
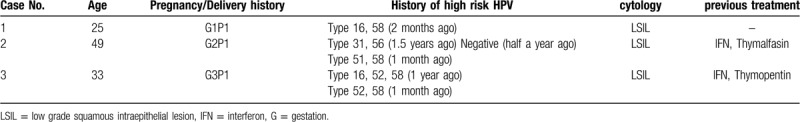
Patient characteristics before receiving hyperthermia treatment.

All patients did not experience any adverse effects during and after the treatment.

## 3 Discussion

With the changing of sexual culture, the incidence rate of uterine cervical neoplasia has been increasing among young women.^[1]^ Eradiation of HPV in the early state of the disease is pivotal in prevention of the progression of cervical neoplasia. In our cases, the patients were completely or partly resolved of high-risk HPV and reversed intraepithelial neoplasia by local hyperthermia treatment. Hyperthermia treatment has shown its effectiveness in HPV infected skin lesions via several mechanisms.^[3–6]^ Depending on our experience, we adopted the same temperature, the same therapeutic course and the same parameters in the eradication of HPV, irrespective of HPV types. HPV may inhibit the activation of cytotoxic T cells, which is critical for eradicating HPV-infected cells. Hyperthermia may promote the release of cytokines and expression of cell surface molecules in dendritic cells, resulting in stimulation of antigen-specific cytotoxic T lymphocytes.^[7]^ Furthermore, hyperthermia may elevate phagocytosis and antigen uptake by macrophages and dendritic cells, promote the migratory maturation of Langerhans cells in HPV-infected skin.^[8,9]^ Regression of HPV-infected lesions after hyperthermia treatment displayed dense infiltration of CD4^+^ and CD8^+^ T lymphocytes.^[6]^ In addition, hyperthermia increased the percentage of apoptotic keratinocytes infected with HPV and cervical cancer cells; induced mutation of HPV DNA sequence and inhibited the virus or virus replication; reduced the copy number of HPV16 E6 in cervical cancer tissue; decreased viral load and viral integration degree.^[10–13]^ It is widely known that the majority of females will harbor HPV at some time in their lives, but in about 2 thirds of women the infection becomes persistent, and the more prolonged the HPV infection, the greater risk of the progression of cervical carcinogenesis.^[14,15]^ Early clearance of HPV relieved the heavy mental burden of the patients. Currently, immunotherapy and other target therapies against HPV infection is emerging.^[16]^ Our cases support the feasibility and acceptability of the use of hyperthermia on the cervix infected with HPV.

There were no serious adverse effects associated with hyperthermia such as heavy vaginal bleeding or burning during treatment and follow-up. Despite incomplete understanding of the mechanism, we consider that the present study may prove beneficial to patients and may be another option to clear HPV when patients fail to respond to other therapies or do not apply other therapies. A randomized controlled trial is required to consolidate the efficacy of thermotherapy on early stage cervical neoplasias with positive high risk HPV.

## Author contributions

**Data curation:** Ruiqun Qi, Xiaodong Li

**Methodology:** Wei Huo, Ruiqun Qi

**Supervision:** Xin Wu, Hongduo Chen

**Validation:** Hongduo Chen, Xinghua Gao

**Writing- original draft:** Yang Yang, Lan Zhang

**Writing- review & editing:** XingHua Gao
